# Survival analysis and prognostic factors of the carcinoma of gallbladder

**DOI:** 10.1186/s12957-022-02857-y

**Published:** 2022-12-20

**Authors:** Zainab Feroz, Priyanka Gautam, Sonia Tiwari, Girish C. Shukla, Munish Kumar

**Affiliations:** 1 Department of Biochemistry, University of Allahabad, Prayagraj, India; 2 Department of Radiation Oncology, Kamala Nehru Memorial Hospital Prayagraj, Prayagraj, India; 3 Department of Biological Sciences, Cleveland State University, Cleveland, USA; 4 Center for Gene Regulation in Health and Disease, Cleveland State University, Cleveland, USA

**Keywords:** Gallbladder cancer (GBC), Prognosis, Overall survival, Prevention

## Abstract

**Background:**

The present study aims to evaluate the survival status of patients with gallbladder cancer (GBC) and explore the prognostic factors for the improvement and preventions.

**Methods:**

The study consists of 176 patients with clinically diagnosed gallbladder cancer; the study was conducted between 2019 and 2021 registered at Kamala Nehru Memorial Cancer Hospital, Prayagraj, India. The survival rates were analyzed by the Kaplan-Meier method; survival rate difference was analyzed by log-rank test, prognosis factors; and hazard ratio for mortality outcomes was estimated using Cox regression method.

**Results:**

The overall median survival time of patients was 5 months with the 1-year, 2-year, and 3-year survival rates of 24.4%, 8.5%, and 4.5%, respectively. The 3-year survival for patients with jaundice was 2.9%, liver infiltration (4.2%), gallstones (0.8%), and with advanced tumor grade (1.4%). Elderly GBC patients had lower survival rates (3.8%), while the 3-year overall survival for patients residing in urban areas dropped to zero. No patients in the tumor stage (T3/T4) and with distance metastasis stage survived in 3 years, while only 1.1% of patients with advanced nodal stage survived. On receiving surgery and radiation therapy, the 3-year survival rate increased to 19.5% and 35%, respectively. The results of multivariate analysis showed that urban region (*HR* = 1.568, *p* = 0.040), gallstone or not (1.571, *p* = 0.049), N stage (*HR* = 1.468, *p* = 0.029), and M stage (*HR* = 2.289, *p* < 0.0001) were independent risk factors for prognosis, while surgery or not (*HR* = 0.573, *p* = 0.030) was the protective factor for the prognosis of GBC.

**Conclusion:**

The overall survival of GBC in the Gangetic belt is poor. The geographical region of patients, gallstones, and N and M stage was the risk factors for prognosis, while surgery or not was the protective factor for the prognosis of GBC.

## Background

Gallbladder cancer (GBC) is an aggressive and highly lethal neoplasm of the biliary tract. Unlike other cancer types, GBC show extreme geographical and ethnic biases, firming its roots particularly in the northeast Indian region where its highest incidence rates had been reported [1]. The North and Northeast Indian population closely follows a high incidence rate of GBC compared to Chile and Bolivia [2, 3]. Within India, GBC segregated the North and South zones, making North, East, Northeast, and Central India as highly prone areas for GBC, while low incidences are found in South and West India [3]. The eastern part of Uttar Pradesh and western Bihar regions near the river Ganges is highest risk regions for GBC [4]. The possible reason for this disparity could be the environmental factors that have a high influence on the etiology of GBC [5]. The studies have shown that GBC etiology is related to carcinogens in the polluted river [6]. The water of river Ganges is a hub of industrial waste effluents [7]. Carcinogens in the river particularly by heavy metals (arsenic, cadmium, chromium, and nickel) and azo dyes are known to elevate cancer risk in this region [8] by causing mutations in oncogenes and tumor suppressor genes [9]. The GBC is associated with other possible risk factors including gallstones, gender, age, obesity, reproductive factors, race, primary sclerosis, cholangitis, gallbladder polyps, congenital biliary cysts, typhoid, *Helicobacter pylori* infection, alcohol intake, smoking, fatty liver disease, unhealthy diet, and environmental exposure to specific chemicals [10]. Since the epidemiology of GBC is highly influenced with the geographical variations and environmental exposures, hence in this study, we have evaluated the possible prognostic factors affecting the survival in the world’s highest risk region of Gangetic belt.

The GLOBOCON 2020 data revealed that GBC account for 84,695 deaths in 2020, which is 0.9% of the global cancer deaths and 115,949 new GBC cases diagnosed in the same year accounting for 0.6% of all the global cancer cases [11]. Management of GBC remains a tedious task due to its unclear and unspecific signs and symptoms. Unfortunately, the symptoms of GBC and gallstone disease share a common ground, resulting in the delayed diagnosis, primarily at its advanced stages [12]. Surgery remains the only potential cure; however, only limited patients diagnosed in their early-stage disease benefit from resection. Radical resection cannot be performed in most advanced staged patient as tumors usually metastasize in adjacent organs [13]. GBC has the shortest median time interval survival [14]. Despite great stride, not much effort is being made to improve the treatment strategy and early detection of the disease. A careful consideration of the epidemiological factors could improve the overall survival (OS) of GBC patients and assist in timely diagnosis. This study aims to evaluate the prognostic factors affecting the survival and to explore the survival status of GBC patients residing in the highest incidence region of the Gangetic belt.

## Methods

**Fig. 1:**
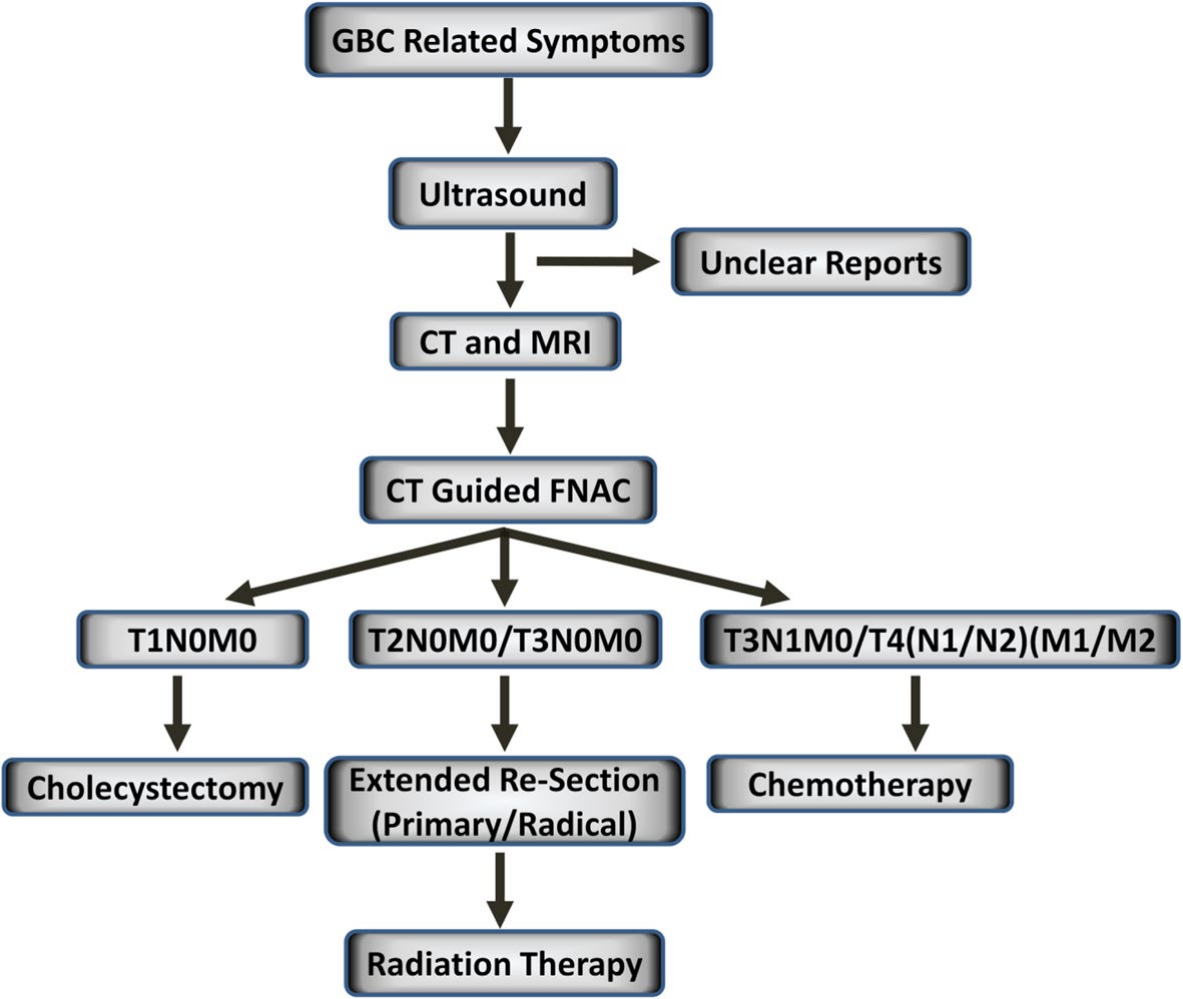
A flowchart illustrating the diagnosis and stage-wise treatment strategy for patients with GBC

We performed a follow-up study; for the study purpose, only those patients with established diagnoses of gallbladder carcinoma were recruited. The study was conducted over a period of 2 years between 2019 and 2021 from Kamala Nehru Memorial Cancer Hospital, Prayagraj, Uttar Pradesh, India. GBC and bile duct cancer are often difficult to distinguish; for accurate diagnosis, physicians at Kamala Nehru Memorial Cancer Hospital first recommend an ultrasonography (US), on examining the complaints of jaundice/unspecific gastrointestinal complaints/other symptoms related to GBC. If US reveals unclear reports, then computed tomography (CT) and MRI are done which provide additional information, and finally, CT-guided fine needle aspiration cytology (FNAC) of gallbladder mass lesions is performed (Fig. 1 illustrates the flowchart representing the diagnosis and stage-wise treatment strategy for patients with GBC).

### Inclusion criteria and exclusion criteria

The inclusion criteria were as follows:

(i) Histopathologically approved GBC patients (those patients whose diagnosis was determined through computed tomography (CT)-guided fine needle aspiration cytology (FNAC) of gallbladder mass lesions) of ethnic North Indian origin.
(ii) Patients within the age range of 18–65 years

The exclusion criteria were as follows:

(i) Patients with an unclear or incomplete diagnosis of cancer
(ii) Patients under the age of 18 years
(iii) Patients above 65 years of age
(iv) Double metastatic and/or multiple source cancer
(v) Patients who died within 1 month of diagnosis
(vi) Patients with incomplete and/or no follow-up information

### Data extraction

Demographic data, clinicopathalogical information, and treatment strategy data were evaluated based on the hospital record of patients at the time of the first diagnosis. The data included patient ID, age, gender, ethnicity, dietary habits, any form of the tobacco intake, the presence of any other chronic disease, jaundice, liver infiltration, gallstones, pathological type, histological grade, gallbladder location of tumor (fundus, body-neck), clinical stage, pTNM stage, treatment strategy (surgery, chemotherapy, radiation), and follow-up (survival month, survival status). The treatment strategy was determined by the physicians as per the patient’s clinical and the TNM stage at the time of diagnosis of the disease. GBC patients diagnosed at the early staged disease (stage 1/2) were recruited for the surgical treatment which was followed by radiation; while the advanced staged patients (stage 3/4) were treated with chemotherapy. Cancer-specific survival was taken as the primary end point.

### Data analysis

The cumulative rate was determined by Kaplan-Meier method, and the difference in survival rate between groups was compared by log-rank test. Cox univariate analysis was performed to calculate the hazard ratio of death. Statistically significant variables from the univariate analysis were selected to carry out the Cox regression model for multivariate analysis through which independent variables for GBC prognosis were determined. In calculating the survival and survival rate, the date at first diagnosis was regarded as the starting date. Overall survival time was determined by the date of cancer diagnosis to the end of follow-up, with the survival status of living or dead. All the tests were conducted at a significance level of 95% with *p* < 0.05 considered statistically significant. All statistical analysis was performed using SPSS software version 16 (SPSS Inc.; Chicago, IL, USA).

## Results

### Baseline characteristics

One-hundred seventy-six GBC cases were recruited in this study from 2019 to 2021. The median age was 53.62 ± 9.560 years old. The percentage of patients within the age range of 45–65 years was 74.4% (131/176). Females comprise 71.6% (126/176) of the total patients, and 84.1% (148/176) of the cases were rural dwellings. About 83% (146/176) followed a vegetarian diet, and 51.1% (90/176) were tobacco consumers (Table 1). The majority of the patients (89.8%, 158/176) were diagnosed with adenocarcinoma. Low/moderately differentiated tumor grade was seen in 59.7% (105/176), with fundus being the precise tumor location (60.2%, 106/176). The percentage of cases that underwent surgery was 24.4% (43/176). The majority of patients received chemotherapy (90.3%, 159/176) with gemcitabine combined with cisplatin as the prime chemotherapy regimen (86.4%, 152/176), while only 9.7% (17/176) of the patients received radiation therapy.

**Table 1:** Baseline and clinicopathalogical characteristics of GBC patients (*N* = 176)

Variable	Item	***N*** (%)
**Age**	26–45	45 (25.6)
	45–65	131 (74.4)
**Gender**	Female	126 (71.6)
	Male	50 (28.4)
**Region**	Rural	148 (84.1)
	Urban	28 (15.9)
**Dietary habit**	Vegetarian	146 (83.0)
	Nonvegetarian	30 (17.0)
**Tobacco chewing**	Non-chewer	90 (51.1)
	Chewer	86 (48.9)
**Jaundice**	Absent	72 (40.9)
	Present	104 (59.1)
**Gallstones**	Absent	51 (29.0)
	Present	125 (71.0)
**Liver infiltration**	Absent	34 (19.3)
	Present	142 (80.7)
**Any chronic disease**	Absent	145 (82.4)
	Present	31 (17.6)
**Histology**	Adenocarcinoma	158 (89.8)
	Non-adenocarcinoma	18 (10.2)
**Grade**	I/II	105 (59.7)
	III/IV	71 (40.3)
**Tumor location**	Fundus	106 (60.2)
	Body-neck	70 (39.8)
**Clinical stage**	I/II	32 (18.2)
	III/IV	144 (81.8)
**T stage**	T1/T2	50 (28.4)
	T3/T4	126 (71.6)
**N stage**	N0	83 (47.2)
	N1/N2	93 (52.8)
**M stage**	M0	68 (38.6)
	M1	108 (61.4)
**Surgery**	No	133 (75.6)
	Yes	43 (24.4)
**Radiation**	No	159 (90.3)
	Yes	17 (9.7)
**Chemotherapy**	No	17 (9.7)
	Yes	159 (90.3)
**Chemotherapy regimens**	Gemcitabine + cisplatin	152 (86.4)
	Gemcitabine + carboplatin	16 (9.1)
	Nill	8 (4.5)

### Survival of patients

**Fig. 2:**
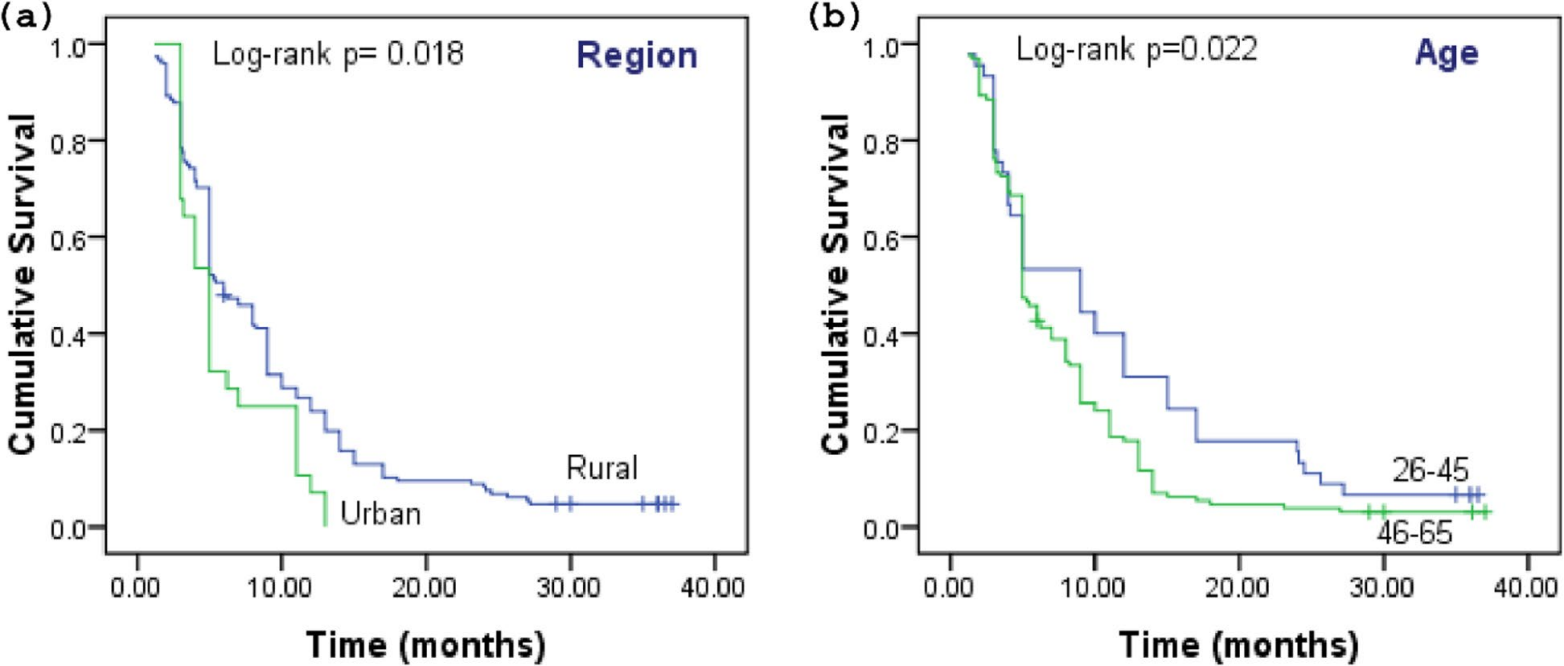
The cumulative survival of GBC patients based on age and region: the figure shows the Kaplan-Meier functions for the survival of GBC patients based on **a** urban vs. rural regions of patients and **b** age groups of 26–45 and 46–65 years. The survival difference was measured by log-rank test

Table 2 shows the 1-year, 2-year, and 3-year survival rates of GBC patients with different groups of categorical variables and results of log-rank test. Results from the Kaplan-Meier function indicate that the survival rates of patients with different categorical variables progressively decrease with the increase in the year, i.e., minimum in the third year. The overall median survival time of all patients was 5 months, and the overall 1-year, 2-year, and 3-year survival rates were 24.4%, 8.5%, and 4.5%, respectively. The 3-year survival of patients with the age range of (46–65) was 3.8%, with a significant difference in the overall survival (OS) (Fig. 2a), while no urban dwelling GBC patient survived in 3 years (Fig. 2b).

**Table 2:** Survival of GBC patients, results of Kaplan-Meier, and log-rank test

Variables	Item	1-year survival rate (%)	2-year survival rate (%)	3-year survival rate (%)	Chi-square	***p***-value, 3-year survival
**Total**		24.4	8.5	4.5		
**Ag**e	26–45	40.0	17.8	6.7	5.215	0.022*
	46–65	19.1	5.3	3.8		
**Gender**	Female	23.0	7.1	4.0	0.012	0.911
	Male	28.0	12.0	6.0		
**Region**	Rural	27.0	10.1	5.4	5.630	0.018*
	Urban	10.7	0.0	0.0		
**Dietary habit**	Vegetarian	25.3	10.3	5.5	1.147	0.284
	Nonvegetarian	20.3	0.0	0.0		
**Tobacco chewing**	Non-chewer	27.2	8.7	2.2	0.080	0.777
	Chewer	21.4	8.3	7.1		
**Jaundice**	Absent	34.7	12.5	6.9	9.385	0.002*
	Present	17.3	5.8	2.9		
**Gallstones**	Absent	58.8	27.5	13.7	33.987	< 0.0001*
	Present	10.4	0.8	0.8		
**Liver infiltration**	Absent	55.9	17.6	5.9	10.837	0.001*
	Present	16.9	6.3	4.2		
**Any chronic disease**	Absent	25.9	9.8	4.9	0.341	0.559
	Present	18.2	3.0	3.0		
**Histology**	Adenocarcinoma	22.8	7.6	5.1	0.202	0.653
	Non-adenocarcinoma	38.9	16.7	0		
**Grade**	I/II	34.3	13.3	6.7	10.149	0.001*
	III/IV	9.9	1.4	1.4		
**Tumor location**	Fundus	28.3	10.4	3.8	1.300	0.254
	Body-neck	18.6	5.7	5.7		
**Clinical stage**	I/II	56.2	18.8	6.2	8.159	0.004*
	III/IV	17.4	6.2	4.2		
**T stage**	T1/T2	54.0	26.0	16.0	29.860	< 0.0001*
	T3/T4	12.7	1.6	0.0		
**N stage**	N0	38.6	14.5	8.4	14.520	< 0.0001*
	N1/N2	11.8	3.2	1.1		
**M stage**	M0	57.4	22.1	11.8	62.191	< 0.0001*
	M1	3.7	0.0	0.0		
**Surgery**	No	13.3	0.0	0.0	39.755	< 0.0001*
	Yes	61.0	36.6	19.5		
**Radiation**	No	19.2	3.8	0.6	23.486	< 0.0001*
	Yes	65.0	45.0	35.0		
**Chemotherapy**	No	18.5	11.1	3.7	0.213	0.645
	Yes	25.5	8.1	4.7		
**Chemotherapy regimens**	Gemcitabine + cisplatin	24.3	7.9	3.9	2.454	0.293
	Gemcitabine + carboplatin	18.8	6.2	6.2		
	Nill	37.5	25.0	12.5		

*all *p*-values <0.05 were considered statistically significant for survival

### Clinical characteristics and survival

**Fig. 3:**
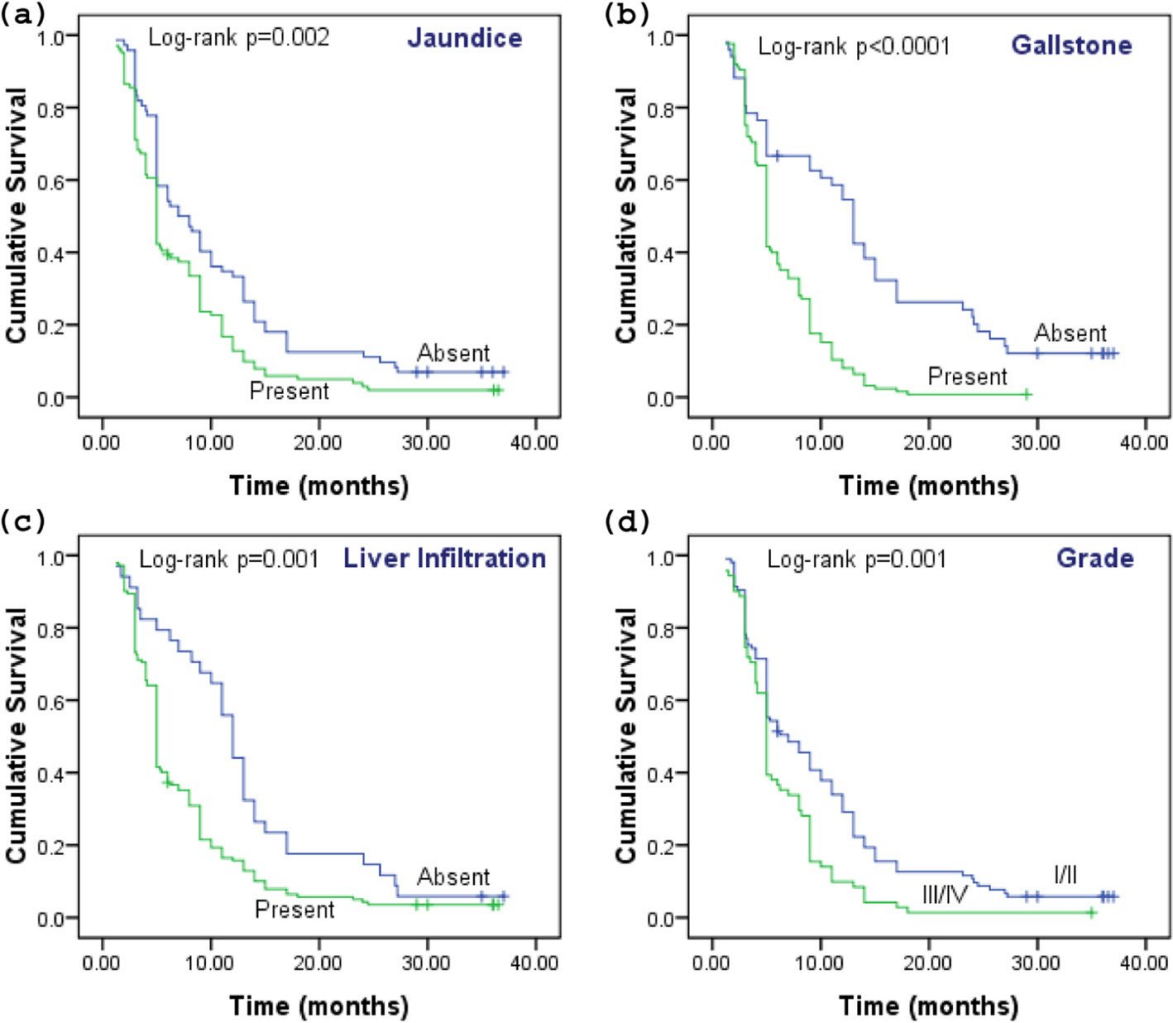
The cumulative survival of GBC patients based on clinical characteristics: Kaplan-Meier functions for the survival of GBC patients based on clinical characteristics including **a** jaundice, **b** gallstone, **c** liver infiltration, and **d** tumor grade. The survival difference was measured by log-rank test

The result of log-rank test shows a significant difference in OS in clinical characteristics. About 2.9% of cases with jaundice survived in 3 years (*χ*^2^ = 9.385, *p* = 0.002) (Fig. 3a). Only 0.8% of GBC patients with complaint of gallstones survived in 3 years (*χ*^2^ = 33.987, *p* < 0.0001) (Fig. 3b), while 4.2% of patients with infiltration of the liver survived in 3 years (*χ*^2^ = 10.837, *p* = 0.001) (Fig. 3c) and with advanced tumor grade 3/4 (1.4%) survived in 3 years (*χ*^2^ = 10.149, *p* = 0.001) (Fig. 3d).

### Stage-wise distribution and survival pattern

**Fig. 4:**
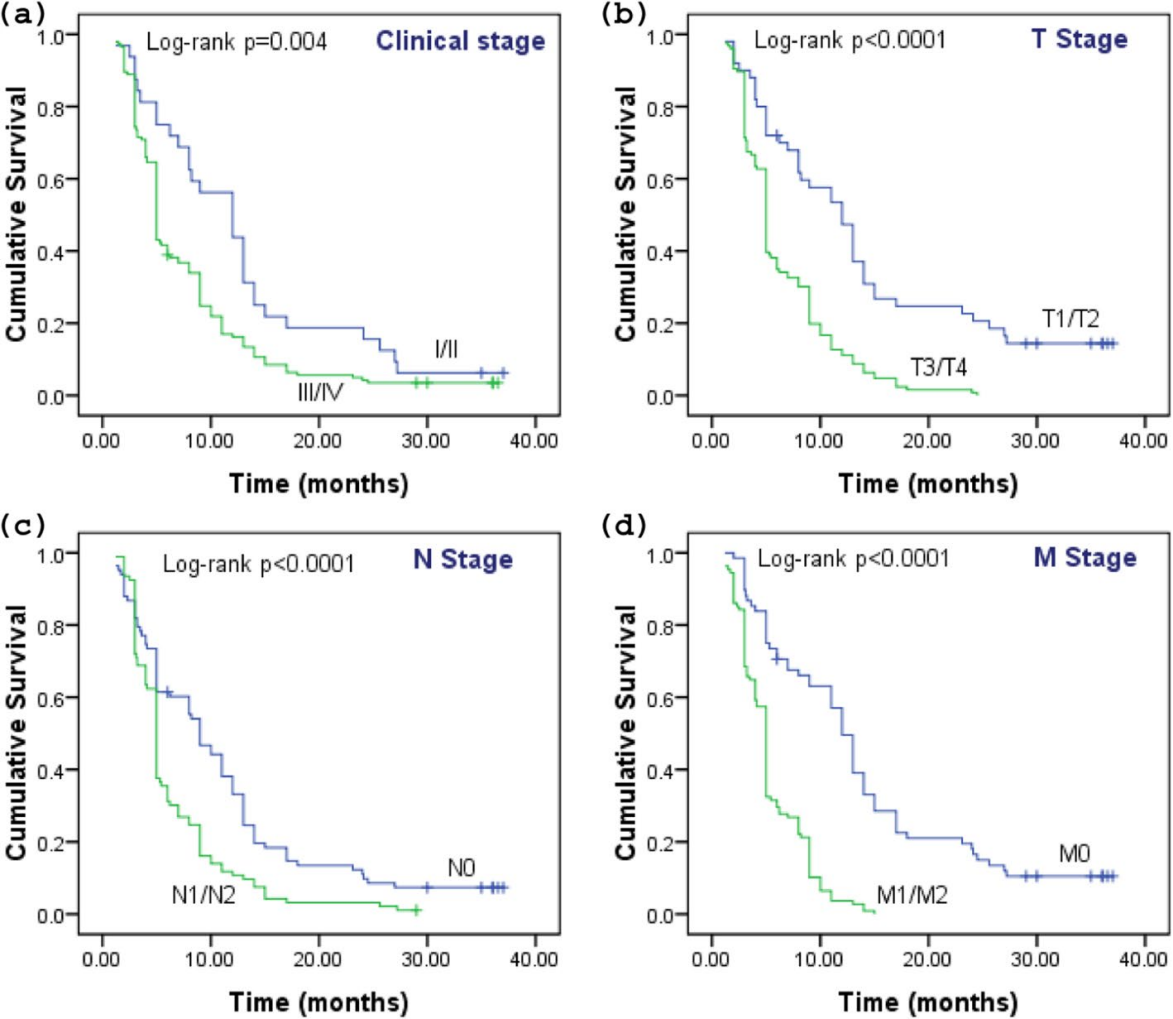
The cumulative survival of GBC patients based on tumor stages: Kaplan-Meier functions for the survival of GBC patients based on **a** clinical stages 1/2 and 3/4, **b** T1/T2 and T3/T4 stages, **c** N0 and N1/N2 stages, and **d** M0 and M1/M2 stages. The survival difference was measured by log-rank test

The estimated 3-year survival for clinical stage 1/2 was 6.2% and for stage 3/4 was 4.2% respectively (Fig. 4a). The 3-year survival for advanced tumor stage is as follows: T1/T2 and T3/T4 were 16.0% and 0.0%, respectively (Fig. 4b); for advanced lymphatic invasion stage—N0 (8.4%) and N1 (1.1%) (Fig. 4c); and for metastatic stage—11.8% of cases with M0 stage survived while no patient in M1 stage (Fig. 4d). The cumulative survival of GBC patients is based on tumor stages: Kaplan-Meier functions for the survival of GBC patients based on (a) clinical stages 1/2 and 3/4, (b) T1/T2 and T3/T4 stages, (c) N0 and N1/N2 stages, and (d) M0 and M1/M2 stages.

### Treatment and survival pattern

**Fig. 5:**
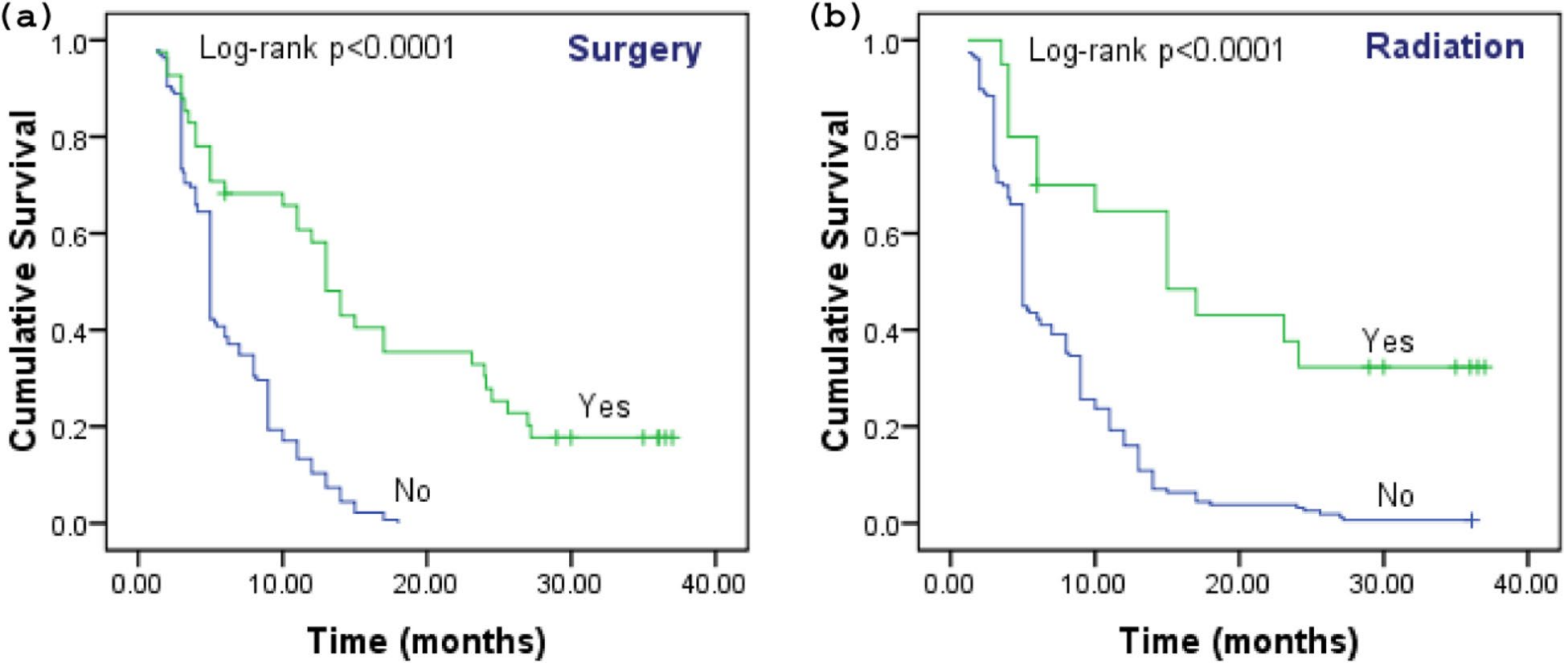
The cumulative survival of GBC patients based on treatments: Kaplan-Meier functions for the survival of GBC patients based on treatment strategy including **a** surgery and **b** radiation. Survival difference was measured by log-rank test

The 3-year survival for patients who underwent surgery was 19.5% (*χ*^2^ = 39.755, *p* < 0.0001) (Fig. 5a), while 35.0% survived when received radiation therapy for treatment (*χ*^2^ = 23.486, *p* < 0.0001) (Fig. 5b). The cumulative survival of GBC patients based on treatments is as follows: Kaplan-Meier functions for the survival of GBC patients based on treatment strategy including (a) surgery and (b) radiation. Survival difference was measured by log-rank test)

### Risk factors associated with prognosis of GBC and OS

Univariate and multivariate proportional hazard regression model was established to analyze the hazard ratio (HR). The results of the univariate analysis showed that increase in patient’s age, urban region-dwelling GBC patients, complain of jaundice, the presence of gallstone, infiltration of liver, advanced tumor grade, advanced clinical stage, and advanced TNM stages were related to the risk factors for the prognosis of GBC; In contrast, surgical treatment and radiation for treatment were related to the protective factors and long-term survival of GBC (Table 3 — univariate Cox regression analysis for prognosis and relative hazard of death in GBC patients).

**Table 3:** Univariate Cox regression analysis for prognosis and relative hazard of death in GBC patients

Variable	Item	HR	95% ***CI***	***p***-value
**Age**	26–45	1	-	Reference
	46–65	1.471	1.029–2.102	0.034*
**Gender**	Female	1	-	Reference
	Male	1.018	0.726–1.427	0.917
**Region**	Rural	1	-	Reference
	Urban	1.590	1.050–2.408	0.028*
**Dietary habit**	Vegetarian	1	-	Reference
	Nonvegetarian	1.224	0.822–1.824	0.320
**Tobacco chewing**	Non-chewer	1	-	Reference
	Chewer	1.402	0.769–1.412	0.792
**Jaundice**	Absent	1	-	Reference
	Present	1.574	1.150–2.155	0.005*
**Gallstones**	Absent	1	-	Reference
	Present	2.778	1.902–4.057	< 0.0001**
**Liver infiltration**	Absent	1	-	Reference
	Present	1.832	1.239–2.710	0.002*
**Any chronic disease**	Absent	1	-	Reference
	Present	1.113	0.756–1.640	0.587
**Histology**	Adenocarcinoma	1	-	Reference
	Non-adenocarcinoma	1.778	1.083–2.917	0.023*
**Grade**	I/II	1	-	Reference
	III/IV	1.604	1.171–2.199	0.003*
**Tumor location**	Fundus	1	-	Reference
	Body-neck	1.183	0.867–1.615	0.289
**Clinical stage**	I/II	1	-	Reference
	III/IV	1.712	1.148–2.555	0.008*
**T stage**	T1/T2	1	-	Reference
	T3/T4	2.583	1.772–3.765	< 0.0001**
**N stage**	N0	1	-	Reference
	N1/N2	1.750	1.280–2.392	< 0.0001**
**M stage**	M0	1	-	Reference
	M1	3.870	2.649–5.652	< 0.0001**
**Surgery**	No	1	-	Reference
	Yes	0.280	0.179–0.438	< 0.0001**
**Radiation**	No	1	-	Reference
	Yes	0.278	0.155–0.499	< 0.0001**
**Chemotherapy**	No	1	-	Reference
	Yes	0.912	0.599–1.388	0.667

*all *p*-values <0.05 were significantly related to the risk factors for prognosis

**more significant compared to the values denoted by single asterisks

To determine the independent risk factors for the prognosis of GBC, a multivariate Cox regression analysis was performed (Table 4). The results revealed that region, gallstones or not, N stage and M stage, and surgery or not were independent factors influencing the prognosis of GBC patients. The urban region, gallstone or not, and N stage and M stage were risk factors for prognosis, while surgery or not was the protective factor for the prognosis.

**Table 4:** Prognostic factors for GBC and results of multivariate Cox regression analysis

Variable	Item	HR	95% ***CI***	***p***-value
**Age**	26–45	1	-	Reference
	46–65	0.955	0.642–1.422	0.822
**Region**	Rural	1	-	Reference
	Urban	1.568	1.020–2.410	0.040*
**Jaundice**	Absent	1	-	Reference
	Present	1.087	0.775–1.523	0.629
**Gallstone**	Absent	1	-	Reference
	Present	1.571	1.002–2.464	0.049*
**Liver infiltration**	Absent	1	-	Reference
	Present	1.448	0.735–2.853	0.285
**Grade**	I/II	1	-	Reference
	III/IV	1.144	0.822–1.591	0.425
**Clinical stage**	I/II	1	-	Reference
	III/IV	0.658	0.300–1.444	0.296
**Tumor stage**	T1/T2	1	-	Reference
	T3/T4	1.794	0.987–3.264	0.055
**N stage**	N0	1	-	Reference
	N1/N2	1.468	1.041–2.070	0.029*
**M stage**	M0	1	-	Reference
	M1	2.289	1.529–3.425	< 0.0001**
**Surgery**	No	1	-	Reference
	Yes	0.573	0.346–0.948	0.030*
**Radiation**	No	1	-	Reference
	Yes	0.573	0.286–1.149	0.117

*all *p*-values <0.05 were considered as the independent risk factors for the prognosis

**more significant compared to the values denoted by single asterisks

## Discussion

GBC is a fatal malignancy of the biliary tract with the shortest median survival duration [15]. In present study, overall median survival was 5 months with 1-, 2-, and 3-year survival rates of 24.4%, 8.5%, and 4.5%, respectively. This result is in agreement with a recent study [16] where 1- and 3-year survival of 29% and 5.4% was observed. Another research reported the OS of 55.5% in the 19-month median follow-up [17]. Differences in the geographical and environmental conditions and the cancer stage of patients could be the primary reason for this disparity.

GBC patients present with advanced stages of regional lymph nodes (N1/N2) and with distance metastasis experience the lowest rates of survival (1.1% and 0.0%, respectively) and have higher mortality risk (*HR* = 1.468 and *HR* = 2.289, respectively). Similar to our result, another research group [18] reported that N and M stages are independent risk factors for GBC. Similarly, other studies reported that the main factor for survival was the stage at diagnosis [14, 19]. The asymptomatic nature of GBC in its early stages causes a delayed clinical presentation relative to the pathological progression resulting in the diagnosis at its advanced stages [20].

Environmental factors play a vital role in GBC susceptibility; hence, careful considerations on environmental risk factors should be made. In this study, we found that the patients residing in urban areas have the significantly low survival rate (0.0% in 3 years) and are at higher risk from GBC mortality (*HR* = 1.568, *p* = 0.040). This is consistent with ecological studies, suggesting that variability in the lifestyle which is important risk factors in the progression of gallstones and GBC. Also, a higher rate of typhoid infection, usage of certain medications, and environmental carcinogens (due to pollution) are more prevalent in urban regions [21], contributing to higher GBC risk in these areas. Consistent with our study, it has been reported that soil in the proximity to the river Ganga has higher arsenic level and therefore may prone for higher GBC in this region [6]. Higher mortality risk and poor survival were observed in older individuals with GBC; increased prevalence of gallstones and higher probability of getting typhoid infection in elderly people might be the probable reason.

Further analysis of the clinical characteristics and the survival of GBC cases revealed the presence of gallstone as an independent risk factor in GBC prognosis. In the present study, the 3-year survival of patients with gallstones was 0.8%, with 1.571 times higher hazard ratio of death, which is consistent with another report indicating that the presence of gallstones is a risk factor for GBC mortality [22]. Additional studies have revealed that the presence of gallstones causes pathological changes in the gallbladder, which might progress to neoplastic changes (epithelial hyperplasia, dysplasia, and metaplasia). Atypical changes in epithelial hyperplasia might lead to the advancement of carcinoma of the gallbladder [23].

The survival analysis by treatment showed that most of the patients received chemotherapy compared to surgical treatment and radiation; however, surgery was the only independent protective factor for GBC. On similar ground, a population-based study conducted in Canada examined the change in treatment modality and trends in GBC survival, where highest 5-year survival was observed for the surgical resection group in 1/2 staged GBC patients [24]. Contradictory to our result, locally advanced cancer was shown to improve survival upon treatment with adjuvant radiation therapy [25]. Surgery is a curative cure for GBC; however, only early staged GBC patients are ideal candidates for surgical treatment, while mostly GBC is diagnosed at an unresectable locally advanced stage [26]; moreover, chances of recurrence remains even after surgical resection [27]. The addition of adjuvant chemotherapy with surgical resection may improve outcomes [28, 29]. Radiation therapy could improve prognosis, especially in the patients with region metastasis, but remains underutilized [30].

Through our work, we have drawn an overview of the impact of GBC on survival and the underlying factors affecting its prognosis in the high- risk area of the North Indian region. The Gangetic belt is one of the regions where incidences of GBC are highest; through this study, we have evaluated the underlying reason behind this disparity. Also, poor prognosis and low survival rates are found in this region; hence, a close consideration on the underlying epidemiologic and clinical factors may assist in early diagnosis and improve the overall survival.

The major strength of our study is that we have recruited those patients only whose complete clinical records were available; hence, a high predictive accuracy was expected. We acknowledge that a minor limitation of our study is that radiation therapy was performed only on the limited number of patients. Moreover, the majority of the patients were in the advanced cancer stage; hence, a concluding remark cannot be made on the effect of surgical treatment on the OS of GBC patients.

## Conclusions

Survival of GBC patient is dismal with poor prognosis, particularly in the Gangetic belt of the North Indian region. The geographical region of patients, gallstones, and N and M stages were linked with the risk factors for prognosis. Surgical treatment is associated with improved OS and serves as the protective factor for the prognosis; however, use of this treatment approach is limited. Improvements need to be made to facilitate the exploration of prevention and early detection of GBC. Advanced treatment strategies and multidisciplinary treatment approach in GBC patients with advanced stage need immediate attention.

## Data Availability

The datasets used and/or analyzed during the current study are available from the corresponding authors on reasonable request.

## Abbreviations

GBC: Gallbladder cancer
OS: Overall survival
HR: Hazard ratio
